# Hyperuricemia Is an Independent Risk Factor for New Onset Micro-Albuminuria in a Middle-Aged and Elderly Population: A Prospective Cohort Study in Taiwan

**DOI:** 10.1371/journal.pone.0061450

**Published:** 2013-04-24

**Authors:** Hung-Yu Chang, Pei-Hsien Lee, Chen-Chou Lei, Chun-Wu Tung, Yung-Chien Hsu, Tung-Jung Huang, Long-chuan Lu, Chun-Liang Lin

**Affiliations:** 1 Department of Nephrology, Chang Gung Memorial Hospital, Chiayi, Taiwan; 2 School of Traditional Chinese Medicine, Chang Gung University College of Medicine, Tao-Yuan, Taiwan; 3 Graduate Institute of Clinical Medical Science, Chang Gung University College of Medicine, Tao-Yuan, Taiwan; 4 Department of pulmonary medicine, Chang Gung Memorial Hospital, Yunlin, Taiwan; 5 Graduate school of Marketing Management, College of Management, National Chung Cheng University, Chiayi, Taiwan; University of Tokushima, Japan

## Abstract

**Background:**

Hyperuricemia is now regarded as a risk factor for cardiovascular disease. Micro-albuminuria is associated with increased risk for cardiovascular disease and chronic kidney disease. We hypothesized that elevated serum uric acid (UA) is associated with development of micro-albuminuria in the general population.

**Methodology/Principal Findings:**

We conducted a community-based prospective cohort study. A total of 1862 subjects from southern Taiwan, all older than 40 years, were screened and 993 of these participants without micro-albuminuria were followed for 4 years. Urinary albumin-to-creatinine ratio was measured two times per year. A multiple linear regression model indicated that serum UA was independently associated with ln(ACR) after adjustment for 8 factors (age, sex, and 6 metabolic metrics) (β = 0.194, *p*<0.01). Logistic regression analysis indicated that each 1 mg/dL increase of UA was associated with a 1.42-fold increased risk of micro-albuminuria after adjustment for the same 8 factors (OR = 1.42, 95% CI: 1.27–1.59, *p*<0.01). A Cox regression model using subjects with serum UA less than 5 mg/dL as reference group indicated higher hazard ratios (HRs) only found in subjects with serum UA more than 7 mg/dL (HR = 3.54, 95% CI: 2.11–5.93, *p*<0.01) and not in subjects with serum UA of 5 to 7 mg/dL (HR = 1.30, 95% CI: 0.82–2.07, *p* = 0.15).

**Conclusion:**

Hyperuricemia is significantly associated with micro-albuminuria in middle-aged and elderly males and females from a general population in Taiwan. Elevated serum UA is an independent predictor for development of micro-albuminuria in this population.

## Introduction

Recent studies have reported that hyperuricemia is a risk factor for development of cardiovascular disease [1]–[4]. Cell biology and animal studies indicated that chronic hyperuricemia can induce vascular smooth muscle cell hyperplasia, activate the local renin-angiotensin system, stimulate inflammatory makers, and cause endothelial dysfunction [5]–[9]. Furthermore, hyperuricemia may be related to the development of chronic kidney disease (CKD). Some studies have proposed that elevated serum uric acid (UA) is an independent risk factor for CKD [10]–[12], but others have concluded that high serum UA is only a consequence of other coexisting risk factors such as hypertension, obesity, dyslipidemia, and insulin resistance [13]. Clinical studies of the relationship of serum UA and development of CKD have yielded inconsistent results and there is controversy about whether UA is an independent risk factor for CKD.

Micro-albuminuria is a marker of endothelial dysfunction and is considered a prognostic marker of kidney damage. Treatment of micro-albuminuria or proteinuria with angiotensin-converting enzyme (ACE) inhibitors or angiotensin receptor blockers (ARBs) may slow the progression of CKD to end-stage renal disease (ESRD). Elevated urinary albumin excretion is associated with a faster decline in renal function, as indicated by measurements of estimated glomerular filtration rate (eGFR) [14]. Micro-albuminuria is also associated with an increased risk of cardiovascular disease in diabetic patients and in the general population [15]. In diabetic patients, the presence of micro-albuminuria predicts overt proteinuria and progression to ESRD [16]. There is extensive literature linking albuminuria with cardiovascular disease and mortality in diabetic and nondiabetic populations [17]–[19].

Our previous study examined the association between hyperuricemia and CKD [20]. Micro-albuminuria is a well-known early marker of CKD. If hyperuricemia is also an independent risk factor for CKD, the causality of hyperuricemia and micro-albuminuria can provide the evidence of hyperuricemia to be the risk factor of CKD. Several measurements provide assessment of overall renal function, such as eGFR and urinary protein [21]. In particular, a decline in eGFR and the development of proteinuria indicate evident renal damage. It is very important to identify a sensitive biomarker for kidney disease before the development of obvious renal damage. Micro-albuminuria may be a good surrogate marker for the onset of kidney damage. Examination of the relationship of serum UA and micro-albuminuria in the general population may help to clarify the role of UA in CKD.

We conducted a prospective cohort study to evaluate the association of hyperuricemia and micro-albuminuria in a population of middle-aged and elderly adults from Taiwan. Our work tried to examine whether hyperuricemia is an independent predictor for development of new-onset micro-albuminuria.

## Materials and Methods

### Participants

This community-based, prospective cohort study of residents of Chiayi County (southern Taiwan) was performed from March 2008 to June 2012. Residents aged 40 years and older were invited to participate. All participants provided written informed consent, and the ethics committee of the Chang Gang Memorial Hospital (CGMH) approved this study (IRB N0 96–1212B).

A total of 1862 subjects aged 40 yrs and older were screened initially. Subjects with micro-albuminuria (measured as described below), who used UA lowering agents, or with CKD stage 3, 4, or 5 (eGFR<60 mL/min/1.73 m^2^) were excluded from follow-up. CKD was classified according to the National Kidney Foundation Kidney Disease Outcomes Quality Initiative (NKF-K/DOQI) guidelines [22]. The abbreviated Modification of Diet in Renal Disease (MDRD) equation [23] was used to estimate GFR (mL/min/1.73 m^2^):

eGFR = 186.3 ×[serum creatinine (mg/dL)]^–1.154^ ×age (years)^−0.203^×(0.742 if female). Participants who reported diabetes mellitus (DM) history, under oral antihyperglycemic agents treatment, hemoglobulin A1C above 6.5% or fasting glucose≧126 mg/dl were regarded having DM [24]. Participants who reported having hypertension (HTN) history or taking antiHTN medication were regarded to have HTN. According to the definition of JNC-6, participants with measured blood pressure more than 140/90 mmHg also were regarded having HTN [25].

A total of 1284 subjects met the criteria for follow-up and were followed for four years. Urinary albumin and urinary creatinine were measured twice a year. Participants who were lost to follow up with missing demographic information or laboratory values were excluded from analysis. Finally, a total of 993 subjects were enrolled for Cox regression model analysis. Four trained assistants used a questionnaire to collect clinical and demographic information, including gender, age, personal history, family history, medical history, medication information (especially the use of angiotensin receptor blocker [ARB] or angiotensin converting enzyme inhibitor [ACEI]) and life style at enrollment. All participants were given medical checkups at enrollment that included physical examinations and laboratory tests.

### Baseline measurements

The clinical examination included measurement of height, weight, blood pressure, and body mass index (BMI) in kg/m^2^. Blood pressure was measured using a mercury sphygmomanometer after rest in a seated position for at least 5 min. Two measurements were made 60 s apart; if the measurements differed by more than 20 mmHg, a third measurement was taken and the 2 closest values were averaged. Serum collection was performed at a mobile examination center. Participants were asked to fast overnight for at least 8 h and plasma glucose, triglycerides, cholesterol, UA, and creatinine were measured with a Technicon RA-1000 analyzer (Bayer Leverkusen, Germany). For urinalysis, participants were instructed to collect at least 5 mL of midstream urine from the initial morning voiding urine.

### Measurement of micro-albuminuria

Urinary creatinine was measured by colorimetry within 6 h after collection in the laboratory center of CGMH (Chiayi branch). Urinary albumin was measured by immunoturbidimetry with traceability to certified reference material (CRM 470) by a portable device (HemoCue® Albumin, Sweden). The urinary albumin-to-creatinine ratio (ACR) was calculated as urinary albumin (mg/L) divided by urine creatinine (mg/dL). Micro-albuminuria was defined as an ACR of 30 to 300 mg/g[26]. Because urine ACR was checked around twice per year, we obtained three consecutive ACR values in around 12 months. The diagnosis of micro-albuminuria was made when two of three samples fell within the micro-albuminuria range. The event date was the first time that micro-albuminuria detected.

### Statistical analysis

Continuous variables are expressed as means±standard deviations (SDs) and categorical variables as percentages. A *p*-value less than 0.05 is considered statistically significant. Independent sample ***t*** *-*tests were used to compare differences between the hyperurcemic and non-hyperuricemic groups and to compare the differences between the 291 unenrolled and 993 enrolled subjects. The distribution of ACR values was skewed, so the natural logarithms (ln) of measured ACR values were used for statistical analyses.

Multivariate linear regression was used to assess the relationship between ln(ACR) and serum UA, with adjustment for numerous confounding factors. Logistic regression analyses were performed to estimate odds ratios (ORs) and 95% confidence intervals (CIs), with micro-albuminuria as a dependent variable and the risk factors and UA level as independent variables. Cumulative incidence of micro-albuminuira was calculated by Kaplan-Meier survival functions. The Cox regression model was used to determine the hazard ratios (HRs) of different uric acid levels in predicting micro-albuminuria after covariates adjusted in all participants and both genders seperately. All statistical calculations were performed using SPSS software version 15.0 for Windows (SPSS Inc., Chicago, IL, USA)

## Results

### Baseline characteristics of participants

Table 1 shows the baseline characteristics of our study subjects. A total of 1862 participants were initially enrolled. Thirty-three subjects with missing data were excluded. Ultimately, 1829 participants (679 males, 1150 females) were included in the analysis. The mean serum UA level was 6.49±1.32 mg/dL in males and 5.54±1.30 mg/dL in females. About 15% of participants had micro-albuminuria (ACR>30 mg/g), about 12% of participants had diabetes mellitus and around 32% of participants had HTN.

**Table 1:** Baseline characteristics of study subjects.

Characteristic	All (n = 1829)	Males (n = 679)	Females (n = 1150)
age, years	66.09±9.61	67.33±9.66	65.36±9.51
SBP, mmHg	135.69±20.39	137.13±19.82	134.84±20.68
DBP, mmHg	81.73±11.24	82.19±11.08	81.46±11.34
BMI, kg/m^2^	24.92±3.51	24.86±3.33	24.95±3.61
cholesterol, mg/dL	204.66±39.10	197.91±39.87	208.64±38.09
triglycerides, mg/dL	125.10±89.16	133.94±118.16	119.88±65.85
fasting glucose, mg/dL	106.87±33.56	108.82±36.72	105.72±31.50
HBA1C,%	6.00±1.12	6.01±1.18	5.99±1.08
uric acid, mg/dL	5.89±1.39	6.49±1.32	5.54±1.30
urinary ACR, mg/g	19.92±36.09	20.17±34.02	19.77±37.27
eGFR, mg/dL/1.73 m^2^	75.22±20.55	72.07±18.52	77.07±21.45
microalbuminuria,%	15.1	16.8	14.7
hypertension,%	31.9	33.1	31.2
diabetes mellitus,%	12.1	13.7	11.2

Note: Conversion factors for international units: cholesterol in mg/dL to mol/L, ×0.0259; triglycerides in mg/dL to mol/L, ×0.0113; fasting glucose in mg/dL to mol/L, ×0.0555; uric acid in mg/dL to mol/L, ×59.48. values expressed as mean±SD for continuous variables and% for categorical variables.

Abbreviations: SBP, systolic blood pressure; DBP, diastolic blood pressure; BMI, body mass index; urinary ACR, urinary albumin-to-creatinine ratio; e-GFR, estimated glomerular filtration rate.

We diagnosed hyperuricemia if serum UA was above 7.5 mg/dL in males and above 6.5 mg/dL in females according to the report of Nutrition and Health Survey in Taiwan implanted between 1993 to 1996 [27]. Males and females with hyperuricemia had higher urinary ACR than those without hyperuricemia. The mean urinary ACR in males was 16.6±28.4 mg/g in those without hyperuricemia and 28.8±42.4 mg/g in those with hyperuricemia (*p*<0.001). The mean urinary ACR in females was 16.8±31.3 mg/g in those without hyperuricemia and 39.3±57.9 mg/g in those with hyperuricemia (*p*<0.001) (Table 2).

**Table 2:** Characteristics of study population according to hyperuricemia status.

	Women (1150)			Men (679)		
	Hyperuricemia (175)			Hyperuricemia (236)		
Characteristics	Yes	No	P	Yes	No	P
Age (yr)	68.4±9.6	64.8±9.4	<0.001	68.2±9.6	66.8±9.6	0.067
SBP (mm Hg)	137.0±24.0	134.4±20.2	0.140	137.8±19.7	136.8±0.2	0.507
DBP (mm Hg)	81.5±12.1	81.4±11.2	0.961	83.0±10.8	81.8±11.4	0.168
Glucose, fasting (mg/dL)	109.0±34.2	105.1±31.1	0.128	107.5±28.1	109.5±0.6	0.507
BMI (kg/m^2^)	26.1±3.85	24.7±3.5	<0.001	25.4±3.5	24.5±3.2	<0.001
Cholesterol (mg/dL)	205.7±41.0	209.1±37.5	0.281	199.8±40.6	196.9±9.5	0.356
TG (mg/dL)	146.5±82.1	115.1±61.4	<0.001	143.8±92.5	128.7±9.7	0.115
HbA1c (%)	6.1±1.0	5.9±1.1	0.246	6.0±1.0	6.01±1.26	0.949
UACR (mg/g)	39.4±57.9	16.8±31.3	<0.001	28.8±42.4	16.6±28.4	<0.001
eGFR mg/dL/1.73 m^2^	65.7±21.0	79.1±20.9	<0.001	64.7±18.7	75.9±17.3	<0.001

Note: Conversion factors for international units as in table 1; Abbreviations: the same as in table 1.

### Role of UA in micro-albuminuria

We analyzed the association of serum UA with micro-albuminuria by multiple linear regression analysis, with UA as the independent variable and ln(ACR) as the dependent variable. The unadjusted regression was significant for all participants (β = 0.204, *p*<0.01), males alone (β = 0.186, *p*<0.01), and females alone (β = 0.246, *p*<0.01). In addition, adjustment for 2 factors (gender and age), and 8 factors (gender, age, diabetes mellitus, hypertension, triglycerides, cholesterol, eGFR, and BMI) also yielded significant correlations for all participants, males alone, and females alone (*p*<0.01 for all). In particular, after adjustment for all 8 factors, there was a significant relationship for all participants (β = 0.194, *p*<0.01), for males alone (β = 0.161, *p*<0.01), and for females alone (β = 0.196, *p*<0.01) (Table 3).

**Table 3:** Standardized coefficient (β) for linear regression of ln(ACR) as a function of uric acid (independent variable).

Model (with all cases)	All (n = 1829)	Men (n = 679)	Women (n = 1150)
Unadjusted	0.204**	0.186**	0.246**
Adjusted for: gender, age	0.226**	0.184**	0.231**
Adjusted for: gender, age, BMI, Cholesterol, TG, eGFR, DM, and HTN	0.194**	0.161**	0.196**

Model (excluding DM cases)	All (n = 1607)	Men (n = 586)	Women (n = 1021)
Unadjusted	0.18**	0.16**	0.23**
Adjusted for: gender, age	0.21**	0.16**	0.22**
Adjusted for: gender, age, BMI, Cholesterol, TG, eGFR, and HTN	0.18**	0.13**	0.20**

Abbreviations: DM, diabetes mellitus; HTN, hypertension; others the same as in table 1

Notes:

a. * denotes *p*<0.05, ** denotes *p*<0.01.

b. ACR was transformed into ln(ACR) before regression analysis for better normality.

c. Gender was removed from the model when data were analyzed by sex.

Table 4 shows the ORs for micro-albuminuria based on multiple logistic regression analysis, with the same adjustments as applied above. As above, the results indicate significant ORs for all participants, males alone, and females alone with no adjustment, adjustment for 2 factors, and all 8 factors (*p*<0.01 for all). In particular, after adjustment for all 8 confounders, each 1 mg/dL increment of UA was associated with a 1.42-fold increased risk of micro-albuminuria in all patients (OR = 1.42, 95% CI: 1.27–1.59, *p*<0.01), a 1.21-fold increased risk in males (OR = 1.21, 95% CI: 1.02–1.44, *p*<0.01) and a 1.57-fold increased risk in females (OR = 1.57, 95% CI: 1.35–1.82, p<0.01).

**Table 4:** Odds ratio for MAU by uric acid treated as a continuous variable.

Model	All (n = 1829)	Men (n = 679)	Women (n = 1150)
Unadjusted	1.47** (1.34∼1.62)	1.29** (1.10∼1.51)	1.64** (1.43∼1.87)
Adjusted for: gender, age	1.48** (1.33∼1.64)	1.30** (1.11∼1.52)	1.60** (1.40∼1.83)
Adjusted for: gender, age, DM, HTN,TG, Chol, eGFR, BMI	1.42** (1.27∼1.59)	1.21** (1.02∼1.44)	1.57** (1.35∼1.82)

Abbreviations: MAU, microalbuminuria; DM,diabetes mellitus; HTN, hypertension; TG, triglycerides; eGFR, estimated glomerular filtration rate; BMI, body mass index.

In addition, gender was removed from the model for analysis by sex.

* denotes *p*<0.05, ** denotes *p*<0.01.

### UA and the risk of development of albuinuria

**Figure 1:**
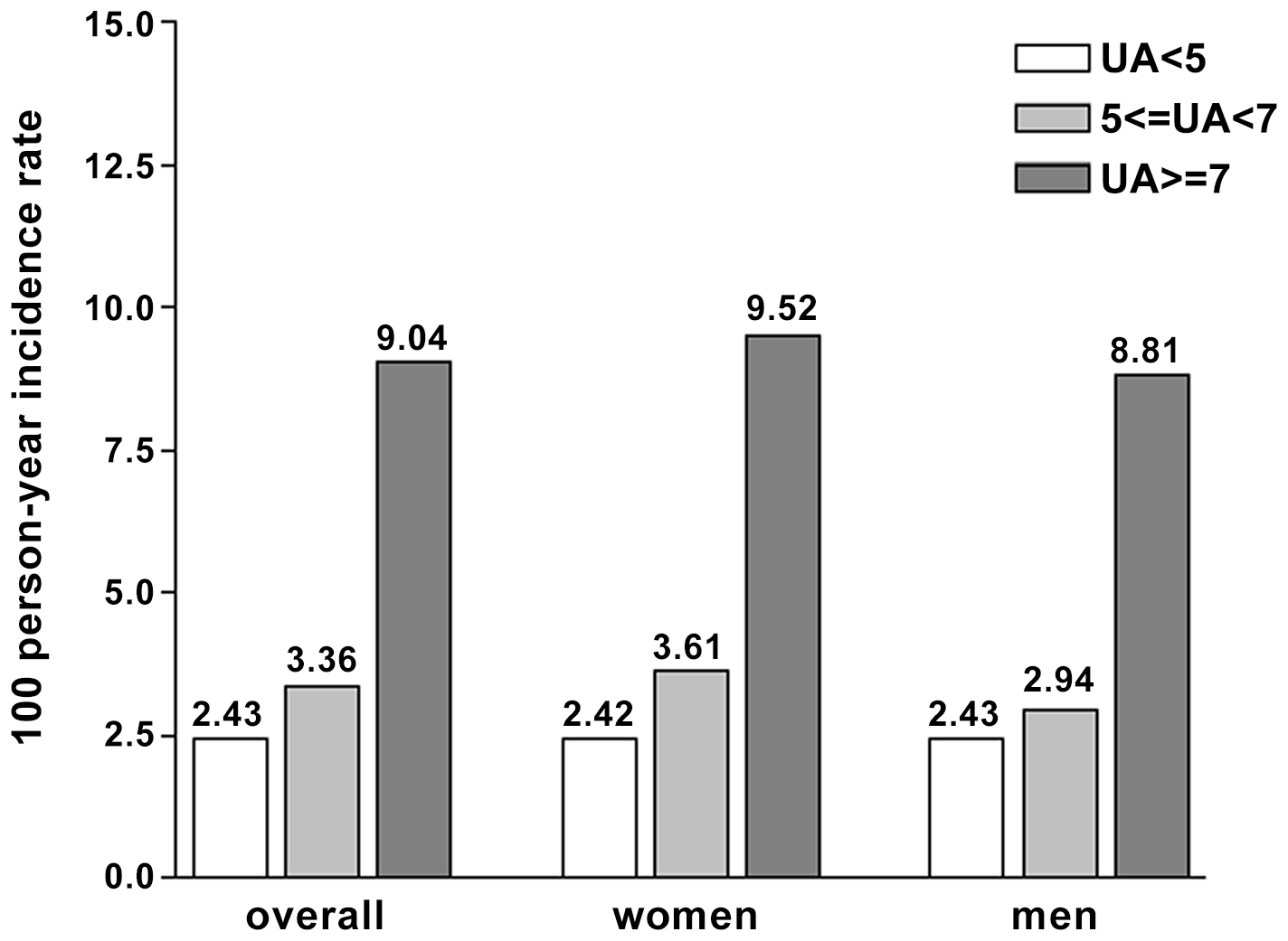
Overall and Sex-specific person year incidence rate of new-onset micro-albuminuria in three serum uric acid tertiles (5 mg/dL or less, 5–7 mg/dL, more than 7 mg/dL) during the four years of follow-up. Conversion factor for uric acid in mg/dL to mol/L, ×59.48.

A total of 545 participants at enrollment had urinary ACR more than 30 mg/g or CKD stage 3, 4, or 5 who were excluded from follow-up. As a result, a total of 1284 participants were eligible for follow-up and 993 participants completed all four years of follow-up. The reasons of loss to follow up in our selected cohort were as following: (1) unwillingness to return for follow-up (N = 195); (2) admission to hospital (N = 36); (3) migration to other county or death (N = 33); (4) taking allopurinol or other uricosuric agents during study period (N = 27). Figure 1 shows the 100 person-years incidence rate of participants with urinary ACR above 30 mg/g in the four years' follow-up for three UA tertiles based on UA at study enrollment (5 or less mg/dL, 5–7 mg/dL, and more than 7 mg/dL). The results indicated that participants with highest serum UA tertile were more likely to have ACR above 30 mg/g during follow-up period.

**Figure 2:**
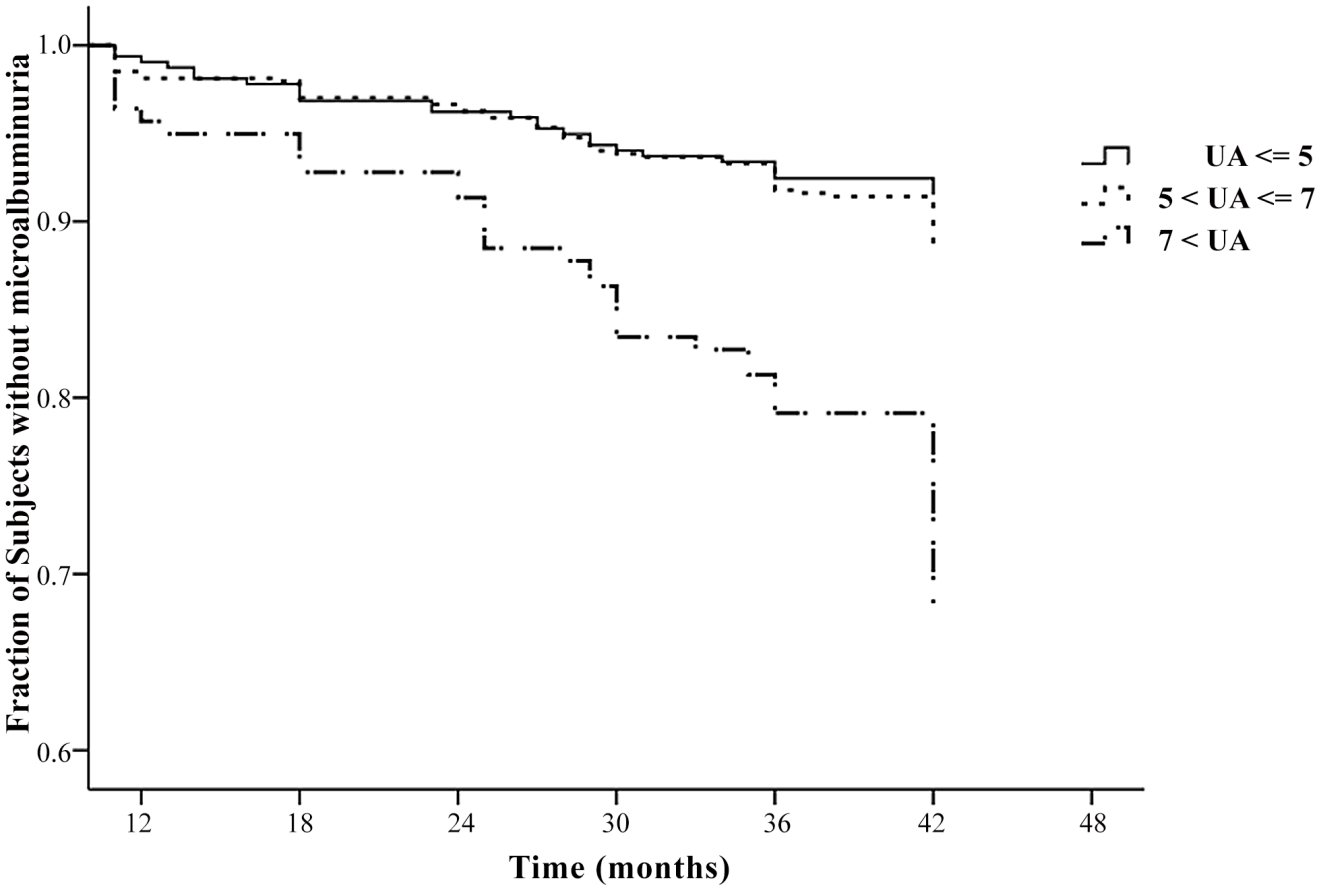
Kaplan-Meier survival analysis for development of micro-albuminuria in patients with different levels of serum uric acid (5 mg/dL or less, 5–7 mg/dL, more than 7 mg/dL). Conversion factor for uric acid in mg/dL to mol/L, ×59.48.

We also performed Kaplan-Meier survival analysis of patients in the same three UA tertiles to assess the risk for development of elevated ACR (Figure 2). Again, the results indicate that micro-albuminuria was more likely to occur in the group with UA level above 7 mg/dl.

Finally, we evaluated the risk of development of micro-albuminuria in subjects with different UA levels by use of a multivariate Cox regression model in all participants and both genders seperately (Table 5). After controlling for all confounders including taking ACEI or ARB as antiHTN medication, participants with serum above 7 mg/dL had higher risk for development of micro-albuminuria after 4 years. Relative to subjects with serum UA less than 5 mg/dL, the HR was 3.54 (95% CI: 2.11–5.93) in the group of UA above 7 mg/dL. The result was statistically comparable in both men and women. However, there was no statistical significance between the two groups of UA level between 5–7 mg/dL and less than 5 mg/dL.

**Table 5:** Hazard Ratios for development of microalbuminuria in different uric acid tertiles by Cox regression analysis (the lowest tertile as reference).

	HR (95% CI)		
	All (N = 993)	Men (N = 345)	Women (N = 648)
UA<5	1	1	1
5≦UA<7	1.30 (0.82∼2.07)	1.40 (0.46∼4.23)	1.35 (0.79∼2.29)
UA≧7	3.54 (2.11∼5.93) **	4.58 (1.50∼14.03)**	3.17 (1.55∼6.48)**

Notes: UA = uric acid (mg/dL).

a. * denotes *p*<0.05, ** denotes *p*<0.01.

b. Adjusted confounders: gender, age, BMI, Cholesterol, TG, eGFR, DM, HTN and with or without ARB, ACEI use.

c. gender was removed from the model for analysis by sex.

### Supplemental analyses

Two supplmental analyses were performed. First we used independent sample T test to exam the differencec between the 291 unenrolled and 993 enrolled subjects. There were no differences in UA level (5.69±1.26 VS 5.59±1.26, *p* = 0.139) and also in other variables between these two groups (data not shown).

Second, after participants with DM excluded, we used multiple linear regression analysis to assess the relationship between albuminuria and serum UA. In linear regression analysis, after adjustment for all confounders, there was a significant relationship for all participants (β = 0.18, *p*<0.01), for males alone (β = 0.13, *p*<0.01), and for females alone (β = 0.20, *p*<0.01) (Table 3).

## Discussion

In this study, we demonstrated a relationship between level of serum UA and urinary ACR in southern Taiwanese adults who were more than 40 years-old. We found a significant association between UA and micro-albuminuria. In particular, the OR for development of micro-albuminuria increased significantly and positively with serum UA, even after adjusting for multiple confounders. During our four-year study period, serum UA level was an independent risk factor for development of micro-albuminuria and this risk was independent of age, gender, and other metabolic factors.

Chen at al. performed a cross-sectional study and reported that metabolic syndrome appeared to be an important factor for development of CKD and micro-albuminuria [28]. Kurella at al. recently reported that metabolic syndrome was independently associated with increased risk for incident CKD during 9 years of follow-up of 10,096 subjects who did not have diabetes at study onset [29]. Previous research suggested that hyperuricemia might have a positive association with metabolic syndrome. Our previous study indicated a significant association between individual components of the metabolic syndrome and serum UA concentration in men and women [20]. Thus, based on these results, one may say uric acid associated with CKD or microalbuminuria through the effect of metabolic syndrome. In the present study, we adjusted for the different components of metabolic syndrome (HTN, serum lipids, DM, and BMI). Our multivariate Cox regression model clearly indicated an independent effect of UA in predicting new onset micro-albuminuria. Thus, the positive predictive role of hyperuricemia for development of micro-albuminuria is independent out of the individual components of metabolic syndrome.

Several hyperuricemia-related mechanisms could lead to subtle kidney injury. For example, hyperuricemia can induce endothelial dysfunction, glomerular hypertrophy, afferent arteriolar wall thickening, vascular smooth muscle hypertrophy, inhibit endothelial nitric oxide production and activation of the renin-angiotensin system. Soluble uric acid has been shown to induce inflammatory pathway activation. In vitro studies have demonstrated that uric acid can increase platelet-derived growth factor-A expression, activate extracellular signal-regulated kinase, cyclo-oxygenase-2, monocyte chemoattractant protein-1 and various nuclear transcription factors which can lead to a chronic inflammatory status [30]. Thus, rats with experimentally induced hyperuricemia developed hypertension and renal injury, such as afferent arteriolopathy, mesangial expansion, glomerular hypertrophy, and albuminuria [8], [9], [31]–[33]. So, there is little doubt that uric acid can induce arteriolopathy in animal model. Is there any evidence in human? Kohagura et al. recently provided evidence that UA associated with renal arteriolar hyalinosis and wall thickening. From the kidney biopsy data in 167 patients with CKD, they observed that the higher level of UA tertile the more severity of renal arteriolar hyalinosis and wall thickening. They concluded that hyperuricemia may be related to renal arteriolar damage in patients with CKD [34].

Micro-albuminuria is regarded as an early sign of subtle kidney damage. For diabetic patients, micro-albuminuria is an earliest clinical manifestation of diabetic nephropathy and also is a critical watershed of chronic kidney disease progression. The best way to assess the role of UA in the pathogenesis of CKD is to determine whether UA level affects the development of micro-albuminuria by longitudinal follow-up. Several studies, including that by Resl et al., have demonstrated an independent association between hyperuricemia and micro-albuminuria in patients with diabetes [35]. Studies of a Korean population demonstrated that serum UA level was strongly associated with micro-albuminuria in patients who had pre-hypertension [36]. The study of Tseng showed that serum UA was independently associated with urinary ACR in Taiwanese patients with type 2 diabetes mellitus [37]. However, no previous prospective cohort studies considered the independent influence of hyperuricemia on micro-albuminuria in the general population. Our study examined the role of hyperuricemia in predicting the development of micro-albuminuria in individuals without CKD and in individuals who did not use UA lowering agents. We found that hyperuricemia can predict the presence of micro-albuminuria in this population. Micro-albuminuria is considered to increase the risk of cardiovascular events. Our study provides evidence of a shared pathogenic mechanism of hyperuricemia and micro-albuminuria. So, it is reasonable to conclude that hyperuricemia may also increase the risk of cardiovascular events via the same pathogenic mechanism.

Concerning the gender difference in the role of hyperuricemia to be the risk factor of cardiovascular disease and CKD, some reports concluded elevated serum UA affected cardiovascular outcome or renal function in females, but not in males [2], [38], [39]. Iseki et al. reported a 7-yr cohort study of Japanese residents in Okinawa which identified uric acid as an independent predictor of chronic kidney disease in women not in men [10]. However, our linear regression results indicated that UA was significantly associated with ln(ACR) in both genders after adjustment for confounding factors (age, DM status, HTN status and other metabolic metrics) (β = 0.61, *p*<0.01 in men; β = 0.196, *p*<0.01 in women). Logistic regression analysis also revealed micro-albuminuria was independently correlated with the baseline UA level in male gender and female gender alike.

The time for hyperuricemia promoting the incident of CKD or predicting cardiovascular events may need decade. For example, the Normative Aging Study examined the relationship between uric acid level and the development of hypertension. It took a mean of 10.3±5.5 years of follow-up to prove the positive predicting role of uric acid in the development of hypertension [40]. Weiner et al. pooled two community-based cohorts, the Atherosclerosis Risks in Communities and the Cardiovascular Health Study. They demonstrated that elevated serum uric acid level is an independent risk factor for development of kidney disease during a follow-up of 8.5±0.9 yrs [11]. However, micro-albuminuria is a very early sign of CKD, so we were able to demonstrate a significant effect of UA in development of micro-albuminuria within 4 years. It is also important to consider the threshold effect of serum UA level. In our Cox regression model, the relative risk was elevated only in the group of highest UA tertile and the same result was found in both genders. The same was also for further adjustment with usage of ACEI or ARB. Our results indicated that patients with low to normal UA level had lower risk of developing renal injury. This finding is in agreement with the finding that using UA-lowering agents can slow the progression of CKD [41], [42]. Therefore, further studies to confirm if using uric acid lowing agents in hyperuricemic patients can diminish urinary albumin excretion are indicated.

In conclusion, this is the first prospective cohort study to demonstrate that the level of serum UA is an independent predictor of micro-albuminuria in a middle-aged and elderly population living in the community. Routine measurement of serum UA may help to identify individuals with increased risk for developing micro-albuminuria and CKD.
